# Characterization of polymorphisms in *CFI* and *ARMS* genes and their association with exudative age-related macular degeneration in Algerian patients

**DOI:** 10.22099/mbrc.2022.43634.1743

**Published:** 2022-06

**Authors:** Ghania Abid, Ahlem Messal, Mohammed Harmel, Aicha Idder, Mostefa Fodil, Faouzia Zemani-Fodil

**Affiliations:** 1 Laboratory of molecular and cellular biology, Université des Sciences et de la Technologie d’Oran Mohamed Boudiaf, USTO-MB, Oran, Algeria; 2 Thematic Agency for Research in Health Sciences ATRSSV, Algeria; 3 Department of Ophthalmology, Hassani Abdelkader Hospital, Sidi Bel Abess, Algeria; 4 Laboratory of medical genetic applied in ophthalmology, « Hammou Boutlilis » Ophthalmology Hospital, Oran, Algeria; 5 BiOSSE (Biology of Organisms: Stress, Health, Environment), Le Mans University, F-72085 Le Mans, France

**Keywords:** Characterisation, CFI, ARMS2, nAMD, Algerians

## Abstract

Increasing evidence shows that polymorphisms in *CFI* and *ARMS2* genes can influence exudative age-related macular degeneration (nAMD) risk. The aim of this study was to assess the role of *CFI* rs10033900 and *ARMS2* rs3750846 polymorphisms in susceptibility to nAMD for the first time in the Algerian population. A total of one hundred twenty four controls and seventy two nAMD cases were included in the present study. Genomic DNA was extracted from venous blood leukocytes. *CFI* rs10033900 and *ARMS2* rs3750846 variants were determined by using the real‑time polymerase chain reaction method. Differences in allele and genotype distribution between the cases and controls were tested with adjustment for age by logistic regression analysis. A stratification of case and control groups by age (<65 or ≥65) and by gender (male and female) was also performed. Statistical analyses were done using SPSS21.0. No statistically significant association was observed between *CFI *rs10033900 and *ARMS2* rs3750846 polymorphisms and nAMD risk (p>0.05 for all comparisons). Stratification by age and gender did not show any significant association between these two polymorphisms and nAMD in a sample of the Algerian population. In our study, *CFI* rs10033900 and *ARMS2* rs3750846 polymorphisms did not predispose alone to nAMD in our population. This study is a contribution to the enrichment of the bank data concerning the *CFI* and *ARMS2* genes, reporting, for the first time, the allelic and genotypic frequencies of these genes polymorphisms characterizing the Algerian population.

## INTRODUCTION

Age-related macular degeneration (AMD) is the principal cause of central visual impairment in the elderly population [1]. In advanced stage, AMD could evolve into exudative AMD, also named neovascular or wet AMD (nAMD), this type of AMD is characterised by the formation of new abnormal blood vessel originating from the choriocapillaris [2]. Various genetic loci have been identified as contributing to nAMD; in recent years, a large genome wide association study (GWAS) reported fifty two independently associated variants covering 34 genetic risk loci, including Complement Factor I (*CFI*), Complement Factor H (*CFH)*, and Age-Related Maculopathy Susceptibility 2 gene (*ARMS2*) [3]. 

CFI is a serine protease produced by hepatocytes, macrophages, endothelial cells, and fibroblasts, and plays an important role in the alternative pathway of complement (APC) [4]. By cleaving and inactivating C3b and C4b, factor I reduces C3 and C5 convertases enzymes formation, limiting otherwise the activation of APC and preventing tissue damage [4]. Wang et al. (2008) suggested that AMD results from an abnormal inflammatory process caused by an unregulated activation of APC [5]. Findings of this study demonstrated that amyloid-β protein found in drusen (subretinal extracellular deposits) adheres to complement factor I (CFI) and reduces its function, leading to low-grade chronic inflammation in subretinal tissues [5]. Several variants in *CFI* gene was reported previously to be associated with AMD [6,7]. Of particular interest, *CFI* rs10033900 polymorphism, located 2781 bp upstream of the 3’ UTR of the complement factor *CFI* gene [6]. It was reported for the first time by Fagerness et al (2008) in a Caucasian cohort (1228 cases/825 controls), in which the most significant association was found with the rs10033900 (p=6.46x10^-8^) [6]. This association was also confirmed in meta-analysis GWAS in a European ancestry sample [8]. In addition, *CFI* rs10033900 was studied in different populations with various results reporting its association with AMD [6, 8–11].

Another risk factor for AMD was *ARMS2* gene, which was identified at the chromosome 10q26 [11]. *ARMS2* gene was reported initially, to encode a mitochondrial protein [12]. However, recent studies, reported that ARMS2 protein may initiates complement activation by binding to human apoptotic and necrotic cells membrane [13]. The polymorphism rs3750846 of *ARMS2* gene was associated with AMD in various populations and was identified among the main polymorphisms in the 10q26 region that increase the risk of late AMD [3, 14, 15]. 

Genetic factors contributing to nAMD were deeply investigated in developed countries. However, there is lack of data reporting their impact in developing countries, especially from Northern Africa. Therefore, we conducted for the first time in a sample of the Algerian population a replication study to analyse the frequency of *CFI* rs10033900 and *ARMS2 *rs3750846 polymorphisms and to evaluate the effect of these two polymorphisms on exudative age related macular degeneration. 

## MATERIALS AND METHODS

In total, One hundred ninety six subjects were enrolled in this case-control study: One hundred twenty four healthy controls and seventy two patients with exudative AMD. Patients were recruited from Hassani Abdelkader Hospital (Sidi Bel Abes, Algeria) whereas control subjects were volunteer’s healthy people. All patients and control subjects enrolled in our study provided informed consent, and the work was approved by the ethics committee at the Algerian Thematic Agency for Research in Health Sciences (The Code of Ethics of the ATRSSV for experiments in humans STCE 164), available on https://atrss.dz/std.php?id=749 . Diagnosis of nAMD was confirmed by an ophthalmologic examination and was made based on best-corrected visual acuity (BCVA) test and Snellen chart, slit-lamp examination, dilated fundus examination, fluorescein angiography (FA), and measurement of central retinal thickness (CRT) using optical coherence tomography (OCT). People over the age of 50 who had been diagnosed with nAMD after undergoing full ocular examinations and had choroidal neovascularization (CNV) in one or both eyes were included in this study. CNV resulting from the other causes and the other retinal disorders were excluded. Subjects over the age of 50 with no clinical evidence of nAMD and free of retinal disorder, or any other ocular pathology were recruited as control samples.

The genomic DNA was extracted from venous blood sample of each patient; using the Salting Out method. *CFI* rs10033900 and *ARMS2* rs3750846 SNPs genotyping was performed using the real‑time polymerase chain reaction method (RT-PCR); SNP Genotyping Assay (CFI rs10033900 Assay ID: C__34681305_20/ARMS2 rs3750846 Assay ID: C_27473788_10 ) were used in an appropriate RT‑PCR mixture containing 2X TaqMan Universal Master Mix (Thermo Fisher Scientific, France), and nuclease‑free water. PCR conditions were as following: 10 min at 95°C; 40 cycles (15 s at 95°C, and 1 min at 60°C). 

 Statistical analysis was performed using the SPSS 21.0 software. *CFI* rs10033900 and *ARMS2* rs3750846 polymorphisms were tested for Hardy–Weinberg analysis. We performed Student’s t-test for numerical variables and Chi-squared test for categorical variables to compare characteristics between nAMD and control groups. The distribution of genotypes and alleles in the nAMD patients and control groups was compared using logistic regression analysis measured by odds ratio (OR) and 95% confidence interval (CI) to estimate the impact of the two studied polymorphisms on the exudative AMD development. Statistical analysis was adjusted by age. Difference was considered significant when P value was less than 0.05. Sample size was determined using Quanto software version 1.2, for an unmatched case-control study with 95% confidence interval and 70 to 80% power.

## RESULTS

Cases and controls characteristics are given in Table 1. A significant difference were observed in age between cases (72.0±9.0 years) and controls (62.8±8.9 years). However, gender did not reveal any significant difference between the two groups (p=0.46) (Table1).

**Table 1:** Baseline characteristics distribution among patients and controls

	**Patients (72)** **n (%)**	**Controls (124) ** **n (%)**	**P-value ** ^a^
Age (mean±SD and range)	72.0±9.0 (50-90)	62.8±8.9 (50-89)	<0.01^b^
Gender Male Female	37 (51.4) 35 (48.6)	55 (44.4) 69 (55.6)	0.46
BMI	26±5 (17-39)	26±4 (16-38)	0.3^b^
Smoking Never Ever	30 (41.7) 42 (58.3)	72 (58.1) 52 (41.9)	0.02*
Iris colour Dark Light NA	40 (55.6) 32 (44.4)	80 (80.0) 20 (20.0) 24	0.04*

^SD^ Standard deviation;^ a ^Chi-square test; ^b:^ t student test; ^* ^p < 0.05. ^NA^ data not available

Genotypes and alleles frequencies of *CFI* rs10033900 and *ARMS2* rs3750846 polymorphisms were conformed to Hardy-Weinberg equilibrium in our population (p>0.05). 

Frequencies of *CFI *rs10033900 and *ARMS2* rs3750846 genotype and allele distribution are presented in Table 2. The *C* risk allele of the *CF*I rs10033900 polymorphism had a frequency of 0.61 in the control group, whereas, the C allele frequency of *ARMS2* rs3750846 polymorphism was 0.24 in the same group. Difference in genotype (CC, CT, and TT) and allele (C/T) frequencies between cases and controls were not statistically significant for either *CFI *rs10033900 polymorphism (alleles: p=0.9, genotypes: p=0.8, dominant model: p=0.7, recessive model: 0.8) or *ARMS2 *rs3750846 polymorphism (alleles: p = 0.8, genotypes: p=0.2, dominant model: p=0.3, recessive model: p=0.07) (Table 2). A tendency of TT genotype of *ARMS2* rs3750846 polymorphism to be linked with exudative AMD development when compared to CC and CT genotype in the recessive model, however this difference were not statistically significant (p=0.07) (Table 2). 

**Table 2:** Genotype and allele frequencies of CFI rs10033900 (C/T) and ARMS2 rs3750846 (C/T) among nAMD patients vs controls

			**Cases** **n (%)**	**Controls** **n (%)**	*P*	**OR 95%[CI]**
***CFI*** ** rs10033900**	**Genotypes**	**TT** **CT** **CC**	09 (12.5) 38 (52.8) 25 (34.7)	17 (13.7) 61 (49.2) 46 (37.1)	**Reference genotype** 0.8 1.1 [0.4-2.6]	
	**Alleles**	**T** **C**	56 (39) 88 (61)	95 (39) 153 (61)	**Reference allele** 0.9 0.9 [0.6-1.4]	
***ARMS2 *** **rs3750846**	**Genotypes**	**TT** **CT** **CC**	34 (47.2) 29 (40,3) 09 (12.5)	75 (60.5) 39 (31,5) 10 (08.1)	**Reference genotype** 0.2 1.5 [0.9-2.4]	
	**Alleles**	**T** **C**	97(68) 47 (32)	189 (76) 59 (24)	**Reference allele** 0.8 1.5 [0.9-2.4]	

For analysing the results of table 2 regarding the age, alleles and genotypes distribution of rs10033900 and rs3750846 polymorphisms were compared between subjects under 65 years age and over 65 years age. Results indicated no significant variation in the alleles and genotypes diversity of these two polymorphisms (Table 3).

**Table 3:** Alleles and genotypes distribution of *CFI* rs10033900 and *ARMS2* rs3750846 polymorphisms based on age

	**<65 years**		***P***	**OR** **[95% CI]**	**≥65 years**		***P***	**OR ** **[95%CI]**
	**Case** ** (%)**	**Control** ** (%)**			**Case** ** (%)**	**Control** ** (%)**		
**rs10033900 (** ***CFI*** **) (C/T)**								

**CC** **CT** **TT** **C** **T**	15 77 08 54 46	40 52 08 66 44	0.2 0.2	0.4 [0.1-5.1] 0.6 [0.3-1.3]	40 47 13 63 37	34 46 20 57 43	0.3 0.3	1.7 [0.6-5] 1.2 [0.8-2.1]
**rs3750846 (** ***ARMS2*** **) (C/T)**								

**TT** **TC** **CC** **C** **T**	69 31 00 15 85	68 29 03 18 82	0.8 0.7	1.03 [0.2-3.7] 1.1 [0.4-3.3]	42 42 16 36 64	53 34 13 30 70	0.5 0.3	1.4 [0.5-4.1] 0.8 [0.4-1.3]

## DISCUSSION

In this study, the *C* risk allele of *CF*I rs10033900 and *ARMS2* rs3750846 SNPs was found in 61% and 24% of our sample respectively. This frequency was comparable with that reported in Europeans such as UK, England, Scotland populations, and in cohort in China, and Southeastern United States [7, 16-18]. However, the *C* allele of *CFI* rs10033900 SNP was twice as high in our population as it was in the Chinese (0.32) and Japanese population (0.39) [19, 20]. In addition, Shin et al. (2021) reported that this allele was detected in 66% of Africans, 53% of Americans, 54% of European, but only in 31% of South Asians, whereas that of *ARMS2* rs3750846 SNP was detected in 26% of Africans, 25% of Americans, 19% of Europeans, suggesting  the existence of genetic differences among racial and ethnic groups, affecting consequently AMD prevalence, that was higher in East Asians than in Europeans due to genetic predisposition in East Asians [21]. 

CFI plays an important role as a regulatory protein in the alternative pathway of complement (APC) by converting C3b and C4b to an inactive form to reduce the C3 and C5 convertases formation, limiting otherwise the activation of APC and preventing tissue damage [4]. Further, Studies, including Wang et al. study, demonstrated a significant association of chromosomal 10q26 genes, especially ARMS2, with AMD, suggesting this gene as the most likely major AMD susceptibility gene [22]. ARMS2 protein was reported to initiates complement activation by binding to human apoptotic and necrotic cells membrane, as consequence, variants affecting this protein may have an impact on dysregulation of the complement system which is a significant driver of AMD pathogenesis [13]. Also, recently, Battu et al (2021) reported that ARMS2 protein levels were significantly higher in the AMD patients than in the control group, practically dry AMD patients, indicating that this protein may be involved in the pathogenesis of AMD [23]. Our results showed that there is no significant association of both *CFI* rs10033900 and *ARMS2* rs3750846 gene polymorphisms with exudative AMD in our study, which is the first to investigate this relationship in an Algerian population. This was consistent with results of Cipriani et al. for the rs10033900 SNP in British (p=0.5) and Scottish group (p=0.7) and of that found in Chinese (p=0.8) and Turkish population (p=0.8) [10, 16, 24]. In contrast, Qian et al. identified this variant to be a risk-increasing factor associated to AMD in Chinese population [19]. Moreover, rs10033900 variant was reported to be a decreased risk factor of nAMD in Chinese (p=0.002) and Japanese population (0.003) [20, 25]. Also, a large GWAS study indicated that ARMS2 rs3750846 SNP increase the risk of AMD in Europeans [3]. Recently, it was also shown in a large European consortium as a highest risk of advanced AMD [15]. Furthermore, in the study of Schick et al. (2020), the minor allele “C” was reported as an increased risk of late AMD (*p*≤0.0006) [14]. The present study had some limitations such as the small sample size and the possibility of other polymorphism risk factors was not included in the study. Therefore, an expanded study is required by incorporating a large sample, and investigating variants in other genes in order to give more evidence of a genetic contribution in the etiology of nAMD. 

Finally, this study is a contribution to the enrichment of the bank data concerning the *CFI *and A*RMS2* gene, reporting, for the first time, the allelic and genotypic frequencies of these genes polymorphisms characterizing the Algerian population.

## Conflict of Interest

The authors report no conflict of interest.

## References

[1] Al-Zamil WM, Yassin SA (2017). Recent developments in age-related macular degeneration: a review. Clin Interv Aging.

[2] Heesterbeek TJ, Lorés-Motta L, Hoyng CB, Lechanteur YTE, den Hollander AI (2020). Risk factors for progression of age-related macular degeneration. Ophthalmic Physiol Opt.

[3] Fritsche LG, Igl W, Bailey JN, Grassmann F, Sengupta S, Bragg-Gresham JL, Burdon KP, Hebbring SJ, Wen C, Gorski M, Kim IK, Cho D, Zack D, Souied E, Scholl HP, Bala E, Lee KE, Hunter DJ, Sardell RJ, Mitchell P, Merriam JE, Cipriani V, Hoffman JD, Schick T, Lechanteur YT, Guymer RH, Johnson MP, Jiang Y, Stanton CM, Buitendijk GH, Zhan X, Kwong AM, Boleda A, Brooks M, Gieser L, Ratnapriya R, Branham KE, Foerster JR, Heckenlively JR, Othman MI, Vote BJ, Liang HH, Souzeau E, McAllister IL, Isaacs T, Hall J, Lake S, Mackey DA, Constable IJ, Craig JE, Kitchner TE, Yang Z, Su Z, Luo H, Chen D, Ouyang H, Flagg K, Lin D, Mao G, Ferreyra H, Stark K, von Strachwitz CN, Wolf A, Brandl C, Rudolph G, Olden M, Morrison MA, Morgan DJ, Schu M, Ahn J, Silvestri G, Tsironi EE, Park KH, Farrer LA, Orlin A, Brucker A, Li M, Curcio CA, Mohand-Saïd S, Sahel JA, Audo I, Benchaboune M, Cree AJ, Rennie CA, Goverdhan SV, Grunin M, Hagbi-Levi S, Campochiaro P, Katsanis N, Holz FG, Blond F, Blanché H, Deleuze JF, Igo RP Jr, Truitt B, Peachey NS, Meuer SM, Myers CE, Moore EL, Klein R, Hauser MA, Postel EA, Courtenay MD, Schwartz SG, Kovach JL, Scott WK, Liew G, Tan AG, Gopinath B, Merriam JC, Smith RT, Khan JC, Shahid H, Moore AT, McGrath JA, Laux R, Brantley MA Jr, Agarwal A, Ersoy L, Caramoy A, Langmann T, Saksens NT, de Jong EK, Hoyng CB, Cain MS, Richardson AJ, Martin TM, Blangero J, Weeks DE, Dhillon B, van Duijn CM, Doheny KF, Romm J, Klaver CC, Hayward C, Gorin MB, Klein ML, Baird PN, den Hollander AI, Fauser S, Yates JR, Allikmets R, Wang JJ, Schaumberg DA, Klein BE, Hagstrom SA, Chowers I, Lotery AJ, Léveillard T, Zhang K, Brilliant MH, Hewitt AW, Swaroop A, Chew EY, Pericak-Vance MA, DeAngelis M, Stambolian D, Haines JL, Iyengar SK, Weber BH, Abecasis GR, Heid IM (2016). A large genome-wide association study of age-related macular degeneration highlights contributions of rare and common variants. Nat Genet.

[4] Khandhadia S, Cipriani V, Yates JR, Lotery AJ (2012). Age-related macular degeneration and the complement system. Immunobiology.

[5] Wang J, Ohno-Matsui K, Yoshida T, Kojima A, Shimada N, Nakahama K, Safranova O, Iwata N, Saido TC, Mochizuki M, Morita I (2008). Altered function of factor I caused by amyloid beta: implication for pathogenesis of age-related macular degeneration from Drusen. J Immunol.

[6] Fagerness JA, Maller JB, Neale BM, Reynolds RC, Daly MJ, Seddon JM (2009). Variation near complement factor I is associated with risk of advanced AMD. Eur J Hum Genet.

[7] Ennis S, Gibson J, Cree AJ, Collins A, Lotery AJ (2010). Support for the involvement of complement factor I in age-related macular degeneration. Eur J Hum Genet.

[8] Yu Y, Bhangale TR, Fagerness J, Ripke S, Thorleifsson G, Tan PL, Souied EH, Richardson AJ, Merriam JE, Buitendijk GH, Reynolds R, Raychaudhuri S, Chin KA, Sobrin L, Evangelou E, Lee PH, Lee AY, Leveziel N, Zack DJ, Campochiaro B, Campochiaro P, Smith RT, Barile GR, Guymer RH, Hogg R, Chakravarthy U, Robman LD, Gustafsson O, Sigurdsson H, Ortmann W, Behrens TW, Stefansson K, Uitterlinden AG, Duijn CMV, Vingerlin RJ, Klaver CCW, Allikmets R, Brantley MA, Baird PN, Katsanis N, Thorsteinsdottir U, Ioannidis JPA, Daly MJ, Graham RR, Seddon JM (2011). Common variants near FRK/COL10A1 and VEGFA are associated with advanced age-related macular degeneration. Hum Mol Genet.

[9] Losonczy G, Vajas A, Takács L, Dzsudzsák E, Fekete A, Márhoffer E, Kardos L, Ajzner E, Hurtado B, de Frutos PG, Berta A, Balogh I (2012). Effect of the Gas6 c 834+7G>A polymorphism and the interaction of known risk factors on AMD pathogenesis in Hungarian patients. PLoS One.

[10] Bezci Aygun F, Kadayıfcılar S, Ozgul RK, Eldem B (2020). Complement factor I gene polymorphism in a Turkish age-related macular degeneration population. Ophthalmologica.

[11] Smailhodzic D, Klaver CC, Klevering BJ, Boon CJ, Groenewoud JM, Kirchhof B, Daha MR, den Hollander AI, Hoyng CB (2012). Risk alleles in CFH and ARMS2 are independently associated with systemic complement activation in age-related macular degeneration. Ophthalmology.

[12] Kanda A, Chen W, Othman M, Branham KE, Brooks M, Khanna R, He S, Lyons R, Abecasis GR, Swaroop A (2007). A variant of mitochondrial protein LOC387715/ARMS2, not HTRA1, is strongly associated with age-related macular degeneration. Proc Natl Acad Sci USA.

[13] Micklisch S, Lin Y, Jacob S, Karlstetter M, Dannhausen K, Dasari P, von der Heide M, Dahse HM, Schmölz L, Grassmann F, Alene M, Fauser S, Neumann H, Lorkowski S, Pauly D, Weber BH, Joussen AM, Langmann T, Zipfel PF, Skerka C (2017). Age-related macular degeneration associated polymorphism rs10490924 in ARMS2 results in deficiency of a complement activator. J Neuroinflammation.

[14] Schick T, Lorés-Motta L, Altay L, Fritsche LG, den Hollander AI, Fauser S (2020). The effect of genetic variants associated with age-related macular degeneration varies with age. Invest Ophthalmol Vis Sci.

[15] Colijn JM, Meester-Smoor M, Verzijden T, de Breuk A, Silva R, Merle BMJ, Cougnard-Grégoire A, Hoyng CB, Fauser S, Coolen A, Garcher CC, Hense HW, Ueffing M, Delcourt C, den Hollander AI, Klaver CCW, Consortium ER (2021). Genetic risk, lifestyle, and age-related macular degeneration in Europe: The EYE-RISK Consortium. Ophthalmology.

[16] Cipriani V, Matharu BK, Khan JC, Shahid H, Hayward C, Wright AF, Armbrecht AM, Dhillon B, Harding SP, Bishop PN, Bunce C, Clayton DG, Moore AT, Yates JRW (2012). No evidence of association between complement factor I genetic variant rs10033900 and age-related macular degeneration. Eur J Hum Genet.

[17] Yang Z, Tong Z, Chen Y, Zeng J, Lu F, Sun X, Zhao C, Wang K, Davey L, Chen H, London N, Muramatsu D, Salasar F, Carmona R, Kasuga D, Wang X, Bedell M, Dixi M, Zhao P, Yang R, Gibbs D, Liu X, Li Y, Li C, Li Y, Campochiaro B, Constantine R, Zack DJ, Campochiaro P, Fu Y, Li DY, Katsanis N, Zhang K (2010). Genetic and functional dissection of HTRA1 and LOC387715 in age-related macular degeneration. PLoS Genet.

[18] Schmidt S, Hauser MA, Scott WK, Postel EA, Agarwal A, Gallins P, Wong F, Chen YS, Spencer K, Schnetz-Boutaud N, Haines JL, Pericak-Vance MA (2006). Cigarette smoking strongly modifies the association of LOC387715 and age-related macular degeneration. Am J Hum Genet.

[19] Qian D, Kan M, Weng X, Huang Y, Zhou C, Yu G, Wang T, Zhou D, Zhang Z, Zhang D, Tang W, Liu Y (2014). Common variant rs10033900 near the complement factor I gene is associated with age-related macular degeneration risk in Han Chinese population. Eur J Hum Genet.

[20] Kondo N, Bessho H, Honda S, Negi A (2010). Additional evidence to support the role of a common variant near the complement factor I gene in susceptibility to age-related macular degeneration. Eur J Hum Genet.

[21] Shin HT, Yoon BW, Seo JH (2021). Comparison of risk allele frequencies of single nucleotide polymorphisms associated with age-related macular degeneration in different ethnic groups. BMC Ophthalmol.

[22] Wang G, Spencer KL, Court BL, Olson LM, Scott WK, Haines JL, Pericak-Vance MA (2009). Localization of age-related macular degeneration-associated ARMS2 in cytosol, not mitochondria. Invest Ophthalmol Vis Sci.

[23] Battu P, Sharma K, Rain M, Singh R, Anand A (2021). Serum levels of ARMS2, COL8A1, RAD51B, and VEGF and their correlations in age-related macular degeneration. Curr Neurovasc Res.

[24] Yang F, Sun Y, Jin Z, Cheng Y, Li S, Bai Y, Huang L, Li X (2014). Complement factor I polymorphism is not associated with neovascular age-related macular degeneration and polypoidal choroidal vasculopathy in a chinese population. Ophthalmologica.

[25] Wu PB, Gu H, Yang XF, Liu NP (2013). Association of single nucleotide polymorphism in complement factor I gene with age-related macular degeneration. Zhonghua Yan Ke Za Zhi.

